# Smartphone Addiction and Health Indicators in Portuguese University Students: The Moderating Role of Physical Activity

**DOI:** 10.3390/healthcare14142152

**Published:** 2026-07-16

**Authors:** Bebiana Sabino, Afonso Caixeiro, Pedro Bento, Luís Murta, André Bento

**Affiliations:** 1 Polytechnic Institute of Beja, Higher School of Education, 7800-295 Beja, Portugal; 22827@stu.ipbeja.pt (A.C.); pbento@ipbeja.pt (P.B.); lmurta@ipbeja.pt (L.M.); andre.bento@ipbeja.pt (A.B.); 2 SPRINT Sport Physical Activity and Health Research & Innovation Center, 7800-295 Beja, Portugal; 3 CHRC, Comprehensive Health Research Centre, Department of Sport and Health, School of Health and Human Development, University of Évora, 7004-516 Évora, Portugal

**Keywords:** smartphone addiction, sitting time, sleep quality, sleep time, physical activity, university, students

## Abstract

**Background/Objectives**: Smartphone addiction has emerged as an important public health concern among university students and has been linked to risky movement patterns, such as physical inactivity, prolonged sitting time, and poor sleep outcomes. The purpose of this study was to examine the relationships between smartphone addiction, sleep quality, sleep duration, sitting time, and physical activity among Portuguese higher education students, as well as to see if physical activity moderates the relationship between smartphone addiction and health-related behaviors. **Methods**: A cross-sectional correlational study included 147 Portuguese higher education students (70.1% male; mean age = 20.11 ± 1.94 years). Data were gathered by an online questionnaire that included the Smartphone Addiction Scale-Short Version (SAS-SV), the International Physical Activity Questionnaire-Short Form (IPAQ-SF), and the Pittsburgh Sleep Quality Index (PSQI-PT). Descriptive statistics, Spearman correlations, Mann–Whitney U tests, and hierarchical multiple linear regression analysis were run. **Results**: The prevalence of smartphone addiction was 22.4%. Smartphone addiction was positively associated with sitting time (r = 0.226, *p* = 0.007) and negatively associated with physical activity (r = −0.169, *p* = 0.047). The link between smartphone addiction and sleep quality (ΔR^2^ = 0.035, *p* = 0.028) and sleep duration (ΔR^2^ = 0.039, *p* = 0.017) was associated with a weaker relationship with physical activity. However, neither interaction survived Bonferroni correction for multiple comparisons (adjusted α = 0.017). **Conclusions**: Smartphone addiction is associated with increased sedentary behavior and decreased physical activity levels. The exploratory moderation pattern observed suggests a potential role of physical activity in the relationship between smartphone addiction and sleep indicators, pending replication in larger and more heterogeneous samples.

## 1. Introduction

Physical activity (PA), sedentary behavior, and sleep are increasingly understood as interdependent components of the 24 h movement behavior continuum. Within a fixed 24 h period, time spent in one behavior necessarily affects the time available for the others, supporting the need to examine these behaviors in an integrated rather than isolated way [1]. This perspective is particularly relevant in young adults, since lifestyle risk behaviors commonly emerge earlier in life, tend to co-occur, and may track into adulthood [2]. University students represent a population undergoing important lifestyle transitions, often characterized by increased autonomy, prolonged sitting, irregular sleep routines, high academic demands and intensive use of digital devices [3,4].

Moreover, multiple lifestyle risk behaviors, including physical inactivity, sedentary behavior and poor sleep, tend to co-occur, reinforcing the need to study movement-related behaviors as part of an integrated health profile [2]. However, whether similar associations are present in university students remains less clear, particularly after the transition from school to higher education [3,4].

Young adults, especially university students, can benefit greatly from PA in terms of their physical [5], mental [6] and social [7] well-being. Additionally, the advantages of PA have an effect on academic life, both in terms of academic achievement [8], and the capacity to handle academic stress [9]. The World Health Organization’s recommendations for physical exercise, which demand for 150 to 300 min of moderate-intensity activity or 75 to 150 min of vigorous-intensity activity per week, are not met by many students despite these advantages [10].

In contrast, students spend a significant amount of time on sedentary activities, with averages ranging from 7.29 to 9.82 h per day [11]. This sedentary time, reflected in the hours spent sitting, has adverse health implications and is related to academic activities, such as study or class time, but also to recreational time. These recreational activities are associated with screen time: whether social media, video games, or watching videos [12].

Sedentary behavior and screen exposure are increasingly relevant when studying movement-related health outcomes, although evidence in young adults remains limited. Much of the available research has focused on children, in whom excessive screen time has been associated with poorer motor outcomes, including lower general motor performance, poorer visual-motor integration, and reduced bilateral coordination [13,14]. These findings suggest that prolonged screen-based behaviors may displace opportunities for movement and reduce exposure to varied motor experiences [13,15], a mechanism that may extend to older populations. In young adults, screen exposure is increasingly dominated by smartphone use, yet the extent to which problematic smartphone use affects movement behaviors in this population remains insufficiently studied [16], highlighting the need for research specifically targeting university students.

Excessive and uncontrollable smartphone use is the hallmark of smartphone addiction (SPA), a behavioral disorder that has detrimental effects on day-to-day functioning, mental health, and general wellbeing. It exhibits characteristics including withdrawal symptoms, tolerance, and compulsive use, and it is similar to other behavioral addictions like internet addiction [17]. Among young adults, smartphone use is one of the most relevant forms of screen exposure and has been associated with a range of negative health outcomes, including musculoskeletal symptoms, lower PA [18] and poorer sleep [17]. In a Portuguese cross-sectional study with university students, 31% of participants presented self-reported SPA and 26% reported chronic spinal pain [3]. Also, a systematic revies study shows there was a negative correlation between smartphone use and PA, with increased usage associated with decreased exercise habits [18].

This excessive smartphone use becomes apparent in the evening and at night, which compromises sleep quality and exacerbates depressive symptoms [19]. Failure to fulfill the required 7 to 9 h of sleep per day for this age group, combined with poor sleep quality among college students, has been linked to poorer academic performance, higher levels of anxiety and depression, and greater impairment of daily functioning [20].

Considering the three behavioral patterns over a 24 h period, as discussed earlier, it appears essential for students to reduce sedentary behavior and increase their level of PA, which will have an impact on sleep quality. Accordingly, the following research hypotheses were formulated:

**H1:**  *Students who report greater smartphone dependence have poorer sleep quality and spend more time sitting.*

**H2:**  *Students who are more physically active have better sleep quality and less smartphone dependence.*

**H3:**  *PA moderates the relationship between SPA and sitting time, sleep and time quality.*

The aim of this study is to determine whether smartphone use negatively affects sleep quality, sleep duration, and sedentary behavior, and to explore how PA might mitigate this relationship.

## 2. Materials and Methods

### 2.1. Design and Participants

A cross-sectional correlational study was conducted. The inclusion criteria for the study were students from schools affiliated with the Beja Polytechnic University who used smartphones and were 18 years of age or older. The sample consisted of 213 college students (65.3% male), aged between 18 and 26. However, for the present study, only complete cases were considered for all variables under analysis, resulting in a sample of 147 students (70.1% male) with a mean age of 20.11 (±1.94). Participants who completed the study were significantly older (*p* = 0.025), and the distribution by sex did not differ significantly (*p* = 0.080).

### 2.2. Instruments and Variables

#### 2.2.1. 24 h Movement Behaviors—Physical Activity, Sedentary Behavior and Sleep

To characterize the level of PA the short version of the International Physical Activity Questionnaire (IPAQ-SF) [21] was used. Also, to characterize sedentary behavior, specifically time spent sitting, the same instrument was used. This instrument comprises 7 items, with open-ended questions on the PA of the last 7 days. The final score can be stratified into categories, classifying populations into high-, moderate-, and low-activity groups [21]. In this study the participants were classified into two PA groups based on this IPAQ-SF three-level classification, with the low and moderate categories combined into a single ‘light-to-moderate’ group to ensure adequate cell size, contrasted against the ‘vigorous’ (high) category. Regarding the sleep variable, students answered the question “How many hours do you usually sleep on average per day?” and sleep quality was assessed through a single item from Portuguese version of Pittsburgh Sleep Quality Index [22]—“During the past month, how would you rate your sleep quality overall”—scored 1 (very poor) to 4 (very good), such that higher scores indicate better perceived sleep quality.

#### 2.2.2. Smartphone Addiction

To assess SPA, the Portuguese short version of the Smartphone Addiction Scale–Short Version (SAS-SV) [23] was used. This instrument comprises 10 self-report items rated on a 6-point Likert scale ranging from 1 (“strongly disagree”) to 6 (“strongly agree”). The scale was originally developed and validated by Kwon, Kim [24], and was subsequently adapted and validated for the Portuguese population [23]. Previous validation studies demonstrated adequate psychometric properties, including good internal consistency (α = 0.86) [23] and high reliability (α = 0.911) [24]. In the present sample the SAS-SV also demonstrated good internal consistency (Cronbach’s α = 0.867), confirming the scale’s reliability in this population.

### 2.3. Procedure

Data collection was conducted electronically via the Limesurvey platform. The sample was obtained by disseminating the study via institutional email. Participants were informed about the study’s objectives, that participation in the survey was voluntary, and that their anonymity and confidentiality would be ensured. Data collection occurred from February 2026 to March 2026. The study complied with the ethical principles outlined in the Declaration of Helsinki and was approved by the Ethics Committee of the Beja Polytechnic Institute (PARECER N.° 1/2026).

### 2.4. Statistical Procedures

Data analysis was performed using IBM SPSS Statistics for Windows, version 29.0 (IBM Corp., Armonk, NY, USA). The significance level was set at *p* < 0.05 for all analyses. Prior to inferential analyses, descriptive statistics were calculated for all variables, including mean, standard deviation, minimum, maximum, and median values.

The normality of the data distribution was tested. As several variables did not meet normality assumptions, non-parametric statistical procedures were adopted. Spearman’s correlations were computed to examine the associations between SPA, sleep quality, sleep duration, sitting time, and PA. 95% CIs computed via bias-corrected and accelerated bootstrap with 5000 samples.

To explore differences according to PA level, participants were categorized into two groups: “light-to-moderate” PA and “vigorous” PA. Group comparisons were conducted using the Mann–Whitney U test.

To test the moderating effect of PA on the relationship between SPA and health indicators (sleep quality, sleep duration, and sitting time), hierarchical multiple linear regression analyses were conducted using standardized variables (z-scores). In Step 1, the main effects of SPA and PA were entered into the model. In Step 2, the interaction term between SPA and PA (SPA × PA) was added to determine whether PA significantly moderated the associations between SPA and the dependent variables. The change in explained variance (ΔR^2^) between steps was used to assess the contribution of the interaction term. Given that three moderation models were tested simultaneously (sleep quality, sleep duration, and sitting time), a Bonferroni correction was applied, adjusting the significance threshold to α=0.017 (i.e., 0.05/3). Accordingly, the moderation analyses should be considered exploratory, as interaction effects were evaluated without an a priori power analysis; post hoc estimates indicate that detecting a ΔR^2^ of 0.035 with 80% power at α=0.05 would require approximately *n* = 230 participants.

Additionally, stratified Spearman correlation analyses were performed separately for each PA group to further interpret the observed interaction effects.

## 3. Results

The descriptive statistics for the variables under study are presented in Table 1. With regard to SPA, according to the cutoff values presented earlier, 22.4% of students are addicted. In the PA classification, “light to moderate” activity was reported by 23.1% of participants, and “vigorous” activity by 76.9%. It should also be noted that 61% of students in this study are enrolled in sports science programs, 32.7% of students reported currently participating in organized sports, and 75.8% have participated in the past.

All research variables underwent Spearman’s correlation analysis (Table 2). SPA and sitting time were found to be positively and statistically significantly correlated (r = 0.226, *p* = 0.007). There was a negative correlation between smartphone dependency and PA (r = −0.169, *p* = 0.047). Additionally, a positive connection (r = 0.328, *p* < 0.001) was seen between sleep quality and sleep duration. These results partially confirm H1, showing that greater smartphone dependence is associated with more time spent sitting, but not with poorer sleep quality. H2 was also partially confirmed, as PA was found to be significantly and negatively correlated with SPA.

Table 3 presents the descriptive statistics and the correlations between SPA and the different outcomes according to PA level. The light-to-moderate PA group (*n* = 34) presented a median of 1413 MET-min/week (1486 ± 1258; range: 0–5238), while the vigorous PA group (*n* = 113) presented a median of 5436 MET-min/week (6284 ± 3038, range: 2118–15,438).

To examine whether PA moderated the relationship between SPA and health indicators, hierarchical multiple regression analyses were conducted using standardized variables (Table 4).

For sleep quality, Step 1 did not explain significant variance (*p* > 0.05). In Step 2, the inclusion of the SPA × PA interaction term produced a significant increase in explained variance (ΔR^2^ = 0.035, F change_(1,143)_ = 4.95, *p* = 0.028), with a positive and significant interaction coefficient (B = 0.120, EP = 0.054, *p* = 0.028) (Table 4). The stratified analysis confirmed the moderation pattern: in the “light to moderate” activity group, smartphone dependence was associated with poorer sleep quality (*p* = 0.001), unlike in the “vigorous” activity group (*p* = 0.803) (Table 3).

For sleep hours, smartphone dependence showed a significant main effect in Step 1 (B = −0.200, EP = 0.074, *p* = 0.008; R^2^ = 0.059, F_(2,144)_ = 4.24, *p* = 0.016). The SPA × PA interaction term in Step 2 was also significant (ΔR^2^ = 0.039, F change_(1,143)_ = 5.86, *p* = 0.017; B = 0.191, EP = 0.079, *p* = 0.017) (Table 4). Stratified correlations reinforce that in the “light to moderate” activity group, smartphone dependence was associated with fewer hours of sleep (*p* = 0.031) (Table 3).

For sitting time, stratified correlations reveal that in the “light-to-moderate” activity group, the association between smartphone dependence and sitting time was statistically significant (*p* = 0.010), whereas in the “vigorous” activity group, the correlation was not statistically significant (*p* = 0.061) (Table 3). At the nominal significance level (*p* < 0.05), the SPA × PA interaction was observed for sleep quality and sleep duration. However, applying a Bonferroni correction for the three simultaneous moderation models tested (adjusted α = 0.017) these interaction effects are no longer statistically significant. Additionally, the light-to-moderate PA subgroup comprised only *n* = 34 participants, which limits the stability of these estimates. H3 is therefore not confirmed in a statistically robust sense; the observed pattern is consistent with a moderating role of PA but should be regarded as exploratory and hypothesis-generating, pending replication in larger samples.

## 4. Discussion

The aim of this cross-sectional study is to determine whether smartphone use negatively affects sleep quality, sleep duration, and sedentary behavior, and to explore how PA might mitigate this relationship. This study contributes to a growing body of research examining the co-occurrence of SPA and health behaviors among university students. Despite the growing recognition of SPA as a public health concern, evidence regarding its concurrent associations with sedentary behavior, sleep, and PA in a single sample remains limited, particularly among the age groups analyzed. The present study aims to address these gaps by comprehensively examining the associations between smartphone addiction and 24 h movement behaviors, and by exploring whether PA moderates these relationships.

There are approximately 16.07 million cell phones in Portugal, for a population of 10.15 million (some Portuguese people own more than one cell phone) [25]. All young students’ everyday lives revolve around technology, particularly the use of cell phones. Its use has a dual nature, in that it serves as an important tool for knowledge acquisition and learning, but it also poses a risk to the well-being of young adults [26,27]. Despite its widely acknowledged benefits, the negative sides of smartphone use, particularly SPA, should not be neglected. This addiction is a disorder characterized by an inability to limit one’s usage of the technology. Those affected endure social, psychological, and health-related challenges [28,29]. The global prevalence of digital addiction was analyzed in a meta-analysis. Across all age groups, the prevalence of SPA was 26.99% [30]. Among university students, however, the literature shows some variability, ranging from 47.5% among students at universities in Saudi Arabia [31] to 67.5% in Thailand [32]. In our study, the prevalence of SPA was lower (22.4%) than that reported in other international studies, but closer to that seen in previous national studies [3].

Students addicted to their smartphones report spending excessive amounts of time on their screens, more than eight hours a day, which contributes to a sedentary lifestyle [31]. Smartphone use is linked to bedtime behaviors or sedentary time [33,34]. This study also found that SPA is associated with increased sedentary time. Students’ health is at risk due to a rise in sedentary behavior associated with SPA. On one hand, there are physical health dangers, such as decreased physical fitness and an increased possibility of developing posture disorders [35]. Mental health issues, such as anxiety and depression, are also exacerbated by sedentary lifestyles [36] and SPA [37].

The literature is consistent in reporting a negative association between smartphone use and PA, as observed in this study [38,39]. Students with high levels of SPA have low levels of PA [40]. In addition, students with high levels of PA show low smartphone dependence [39]. In the present study, this association was statistically significant but, the upper bound of the 95% bootstrap confidence interval approached zero, indicating that while real, this effect is small and should be interpreted with caution. Low levels of PA in this demographic are concerning, not only because of their impact on young people’s physical, mental [41] and social [42] health, but also because of the possible negative impacts on academic performance [43].

SPA can impact sleep in addition to the two 24 h movement patterns previously examined. Overuse of smartphones is associated with decreased sleep efficiency, longer wakefulness after falling asleep, and generally worse sleep outcomes [44,45]. This disparity might be explained by the high levels of PA in our group, perhaps as a result of the large percentage of students enrolled in sports science programs. Given that higher levels of physical exercise are associated with better sleep [44].

As a result, the findings suggest that among less physically active students, SPA showed stronger associations with sleep outcomes and sitting time, although these relationships were not statistically significant in the full sample. This pattern is consistent with previous research suggesting that higher PA levels may be associated with attenuated relationships between SPA and sleep outcomes [45,46]. However, these findings should be interpreted cautiously, as 76.9% of participants were classified as vigorous and 61% were enrolled in sports-science programs, meaning the comparison reflects a contrast between moderately and exceptionally active students rather than active versus sedentary individuals. PA improves psychological resilience, self-esteem, and self-control, which may be associated with lower smartphone reliance [45,46]. The focus on promoting active behaviors is crucial in this community, not only because of its association with lower smartphone dependence, but also because it improves academic performance [47] and anxiety [48]. In this regard, the development of structured programs, recreational activities, and participation in sports can be a strategy to employ in higher education, which have proven beneficial in reducing SPA [49]. In addition, digital literacy and mental health programs should be implemented in this context to encourage self-regulation in the use of these devices [50].

One of the study’s limitations is its cross-sectional design, which makes it impossible to demonstrate causality. For instance, a study by Elhai, Dvorak [51] studying the connection between smartphone reliance and sleep reveals that smartphone use is frequently an emotional tactic to counteract sleep disruptions, suggesting that these impacts could only be found through long-term investigations.

Even with validated tools, direct methods could take the place of self-report instruments, especially when it comes to 24 h activity. Additionally, a structural constraint of the study is the sample’s skewed distribution with regard to PA levels. This restricts the representativeness of the sample to physically active university students and limits generalizability to broader or more sedentary student populations. Future research might benefit from replicating the study in samples with varying levels of PA, because in this study the subgroup “light-to-moderate” comprised only *n* = 34 participants and limiting the statistical power of the stratified analyses. An analysis based on the students’ gender or the topic of study they are pursuing may be taken into consideration in further research involving this demographic. Also, sitting time was assessed using a single IPAQ-SF item, which may not accurately capture total sedentary behavior across different contexts. Self-report measures are sensitive to social desirability bias, which occurs when people underreport sedentary behavior or smartphone use while overreporting PA in order to conform to perceived social standards. This is especially important in a sports science sample, where healthy conduct may be highly prized. Another limitation concerns the SAS-SV scale. The cutoff scores used to classify participants as dependent were derived from a validation sample consisting of adolescents with a mean age of 14.5 years [24], which is younger than the participants in the present study. Furthermore, the Portuguese psychometric adaptation of the SAS-SV [23] was published in non-indexed conference proceedings, which limits the strength of the psychometric evidence for this population. In this sense, the SAS-SV’s reliability was independently confirmed in the current sample, indicating good internal consistency.

Despite the limitations outlined above, this study highlights the importance of PA in moderating the relationship between SPA and sleep indicators among college students. In this regard, promoting PA may be essential in university settings, not only for its direct benefits but also for its indirect ones.

## 5. Conclusions

According to the study’s findings, SPA is linked to fewer healthful habits, namely increased sedentary behavior and decreased PA. It is noteworthy that students in the “vigorous” activity group showed weaker associations between smartphone addiction and sleep outcomes, a pattern that warrants further investigation in samples with greater variability in PA levels. Therefore, encouraging PA in academic environments may be a key tactic for reducing the detrimental effects of SPA use in this population.

## Figures and Tables

**Table 1:** Descriptive statistics for the variables under study (*n* = 147).

Variable	Mean	SD	Min	Max	Median
Smartphone Addiction	26.49	9.23	10.0	60.00	26.00
Sleep Quality	2.95	0.60	1	4	3.00
Sleep hours (h)	7.54	0.82	5	10	8.00
Sitting Time (min/day)	344.90	151.12	10	720	360.00
Total PA (MET-min/week)	5174.51	3400.11	0.0	15,438.0	4800.00

SD—Standard deviation; Min—Minimum; Max—maximum.

**Table 2:** Spearman’s correlations between the variables under study (*n* = 147).

Variable	1	2	3	4
1. Smartphone Addiction	—			
2. Sleep Quality	−0.111 [−0.273, 0.063]	—		
3. Sleep hours	−0.157 [−0.307, 0.016]	0.328 ** [0.130, 0.464]	—	
4. Sitting Time	0.226 ** [0.052, 0.385]	−0.231 ** [−0.398, −0.064]	−0.089 [−0.253, 0.083]	—
5. Total PA	−0.169 * [−0.341, −0.006]	−0.104 [−0.227, 0.107]	0.021 [−0.109, 0.207]	−0.129 [−0.288, 0.044]

* *p* < 0.05; ** *p* < 0.01.

**Table 3:** Descriptive statistics by PA group (light-to-moderate vs. vigorous), Mann–Whitney group comparisons, and stratified Spearman correlations between SPA and health indicators within each group (*n* = 147).

	“Light to Moderate” PA		Vigorous PA		Mann–Whitney U
	Mean	SD	Mean	SD	*p*
Smartphone Addiction	27.07	8.14	25.84	8.99	0.319
Sleep Quality (1–4)	3.03	0.50	2.93	0.62	0.398
Sleep hours (h)	7.72	1.00	7.52	0.86	0.470
Sitting Time (min/day)	352.07	153.58	336.36	149.63	0.622
r SPA × sleep quality	−0.561 **		−0.024		
r SPA × Sleep hours	−0.402 *		−0.111		
r SPA × Sitting Time	0.472 **		0.179		

SD—standard deviation; *p*—significance level; r—Spearman correlation; * *p* < 0.05; ** *p* < 0.01.

**Table 4:** Hierarchical regression: the moderating effect of PA on the relationship between SPA and health indicators.

	Sleep Quality			Sleep Hours			Sitting Time		
Predictor	B	SE	*p*	B	SE	*p*	B	SE	*p*
Step 1									
Smartphone Addiction	−0.057	0.051	0.260	−0.200	0.074	0.008 **	22.049	12.745	0.086
PA	−0.047	0.051	0.355	−0.095	0.074	0.202	2.768	12.745	0.828
R^2^	0.015			0.059			0.022		
F	1.01, *p* = 0.366			4.24, *p* = 0.016 *			1.50, *p* = 0.226		
Step 2									
SPA × PA (interaction)	0.120	0.054	0.028 *	0.191	0.079	0.017 *	−19.051	13.666	0.166
R^2^	0.050			0.098			0.036		
ΔR^2^	0.035			0.039			0.014		
F change	4.95, *p* = 0.028 *			5.86, *p* = 0.017 *			1.94, *p* = 0.166		

* *p* < 0.05; ** *p* < 0.01.

## Data Availability

The data presented in this study are available on request from the corresponding author. The data are not publicly available due to privacy and ethical restrictions.

## Abbreviations

The following abbreviations are used in this manuscript:

PA	Physical Activity
SPA	Smartphone Addiction
